# Neural Correlates of Sensory Eye Dominance in Human Visual White Matter Tracts

**DOI:** 10.1523/ENEURO.0232-22.2022

**Published:** 2022-11-18

**Authors:** Ailene Y. C. Chan, Dorita H. F. Chang

**Affiliations:** 1 Department of Psychology, The University of Hong Kong, Hong Kong; 2 The State Key Laboratory of Brain and Cognitive Sciences, The University of Hong Kong, Hong Kong

**Keywords:** binocular vision, eye dominance, imaging, stereovision, white matter

## Abstract

A significant proportion of the human neurotypical population exhibits some degree of sensory eye dominance (SED), referring to the brain’s preferential processing of one eye’s input versus another. The neural substrates underlying this functional imbalance are not well known. Here, we investigated the relationship between visual white matter tract properties and SED in the human neurotypical population. Observers’ performance on two commonly used dichoptic tasks were used to index SED, along with performance on a third task to address a functional implication of binocular imbalance: stereovision. We show that diffusivity metrics of the optic radiations (ORs) well predict behavioral SED metrics. We found no relationship between SED and stereosensitivity. Our data suggest that SED is not simply reflected by gray matter structural and functional alterations, as often suggested, but relates, at least in part to the microstructural properties of thalamocortical white matter.

## Significance Statement

Sensory eye dominance (SED) is a prominent feature of certain clinical populations (i.e., amblyopia). Binocularly neurotypical individuals also have varying extents of SED, with a significant minority exhibiting strong dominance for one eye. We show here that SED in neurotypical individuals is well-predicted by white matter microstructural properties of the optic radiations (ORs). Identifying the neural loci of binocular visual mechanisms allows for targeted paradigms to be developed for shifting eye dominance in the visually impaired.

## Introduction

The visual system relies on integrating information from both eyes to carry out binocular functions such as stereopsis. Information received by the brain via each eye may not be processed with the same weighting, however. Sensory eye dominance (SED) is typically presented as a product of neuroatypical visual development, leading to physiological alterations that impair vision in the weak eye and a tendency of the brain to prefer information obtained by the strong eye (McKee et al., 2003; Levi, 2020). While SED is a prominent feature of certain clinical groups (e.g., amblyopes), SED also exists in a varying extents among binocularly neurotypical individuals, with a significant minority (∼39%) exhibiting strong dominance (Li et al., 2010; Yang et al., 2010).

The mechanistic origins of SED remain difficult to unravel as it has been indexed using behavioral measures involving a variety of visual features and task types. SED is commonly indexed by binocular phase combination (Ding and Sperling, 2006; Zhou et al., 2013), binocular rivalry (Levelt, 1965; Blake, 1989), and more recently, dichoptic signal-in-noise (SNR) tasks (Li et al., 2010; Kam and Chang, 2021). Binocular phase combination tasks present slightly different gratings to each eye and typically allow the observer to adjust the phase, or contrast of the inputs, thereby revealing the relative contribution of each eye’s input to the fused percept (i.e., by indexing a contrast ratio and/or relative phase shift between the two eyes). Binocular rivalry reflects the competition between the two eyes’ signals for visual awareness, quantified by the duration of periods of dominance for each incompatible half-image. Dichoptic signal-in-noise tasks require observers to suppress noise information presented to one eye and extract useful information (signal) presented to the other eye. The dichoptic motion SNR task appears to be a sensitive metric for measuring changes in SED after perceptual training (Kam and Chang, 2021). Although these tasks purportedly all reflect SED, it is unclear as to whether they reflect the same mechanisms.

Models of binocular interactions based on data obtained using binocular phase combination protocols have proposed two stages of binocular contrast gain control: an early stage of interocular suppression (Ding and Sperling, 2006; Baker and Meese, 2007), followed by a second stage of contrast gain control postsummation. These models imply that SED is mediated by processes across a distributed intercortical network. This notion is somewhat consistent with neurophysiological data obtained while using rivalrous stimuli that have shown fMRI responses in the primary visual cortex (V1) and the lateral geniculate nucleus (LGN) to closely couple with perceptual switches (Polonsky et al., 2000; Haynes et al., 2005; Moutoussis et al., 2005; Wunderlich et al., 2005). Further upstream, it has been shown that short-term monocular deprivation in healthy adults appears to modulate activity in V1, as well as in extrastriate visual areas V3 and V4, within which neurons are predominantly binocular (Binda et al., 2018).

While much of the focus on understanding the neural underpinnings of binocular function, and in particular, SED, have focused on gray matter function (Haynes et al., 2005; Wunderlich et al., 2005; Harauzov et al., 2010; H Xu et al., 2016; Binda et al., 2018; K Dougherty et al., 2019), it is entirely possible that SED arises from changes in the efficiency of signal transmission along the visual pathway (i.e., weakened white matter microstructural properties). A potential role of white matter microstructural properties to serving binocular functions has received very little attention, despite the bits and pieces of data available in the literature that suggest they deserve attention. Namely, the optic radiations (OR) between the LGN to V1, have been shown to exhibit greater diffusivity in patients with strabismic amblyopia as compared with healthy controls (Allen et al., 2014; Duan et al., 2015). In addition, left-right asymmetries in diffusion indices in the OR have been found in participants with monocular enucleation as well as in binocular control participants (when comparing contralateral versus ipsilateral tracts to the nondominant eye (Wong et al., 2018). Although each optic radiation does not strictly carry information from one eye only, physiological evidence suggests that V1 responses are largely monocular, and ocular “preference” is preserved beyond layer 4 (Hubel and Wiesel, 1968, 1972; Blasdel and Fitzpatrick, 1984; Douglas et al., 1989; Casagrande and Boyd, 1996). Preservation of eye dominance well beyond known sites of binocular combination could originate from differences in ganglion cell densities. Postmortem analyses on human retinae have revealed an asymmetry in ganglion cells densities, where nasal fibers (i.e., those that eventually decussate) are three times more dense than temporal fibers (Curcio and Allen, 1990). That is, beyond the chiasm then, and within each hemisphere, there is a physiological imbalance as to the white matter representations in terms of the eye of origin. Together, these data suggest the potential for differential monocularly-influenced (albeit not segregated) responses beyond the point of both fiber and gray-matter binocular combination (i.e., beyond the thalamus). Interestingly, while not reflecting eye dominance per se, we note that the posterior portion of the corpus callosum (occipital corpus callosum; OCC) which connects left and right visual cortices, and the vertical occipital fasciculus (VOF), the only currently known long-range tract connecting dorsal and ventral visual areas, have been recently shown to be related to stereoacuity (Oishi et al., 2018).

Here, we asked whether individual variabilities in SED in the neurotypical population can be explained by the microstructural properties of visual white matter tracts along the visual cascade by using diffusion-weighted imaging (DWI). We indexed SED by using two commonly employed tasks: the binocular phase combination and dichoptic motion SNR tasks. We included a third task, depth SNR task to measure depth sensitivity, as it is a functional product of binocular integration (Dieter et al., 2017; Han et al., 2018). Because of low signal integrity for images obtained close to the orbital socket (and nasal cavity), we measured tracts of interest that fell between the thalamic LGN to cortex but did not include the chiasm or optic nerves. Our tracts of interest included: left and right optic radiations, occipital corpus callosum, and left and right vertical occipital fasciculus. Tract tissue properties was indexed using fractional anisotropy (FA) and mean diffusivity (MD). FA quantifies coherence of water diffusion, where higher FA values indicate greater white matter microstructural properties in terms of axon diameter, myelination, etc. (Alexander et al., 2011). MD measures the average rate of water diffusion where lower MD values reflect greater tissue density and/or increased myelination. FA and MD values in visual white matter tracts have been shown to correlate with visual performance and they differ substantially between clinical, e.g., strabismic amblyopia and healthy populations (Thomas et al., 2009; de Blank et al., 2013; Duan et al., 2015; Oishi et al., 2018).

## Materials and Methods

### Participants

A total of 41 observers [mean age: 22.2 (18–28); 21 females] participated in this study. Our final recruitment size was determined via a power analysis following large effect sizes obtained by a previous study investigating the functional (stereoscopic) importance of visual white matter tracts (Oishi et al., 2018). All except for one (an author) were naive to the purpose of this study and all gave written consent in compliance with the study protocol approved by the ethics committee of the Human Research Ethics Committee, The University of Hong Kong. All observers were screened for MRI contraindications, and for neurotypical visual acuity (Sloan LogMar chart, 20/20–2), neurotypical binocular fusion (Worth four dot test) and neurotypical stereoacuity (Titmus test, ≤40 arcs).

### Apparatus and general procedures

Stimuli were generated in MATLAB (The MathWorks) with extensions from the Psychophysics Toolbox (Brainard, 1997; Pelli, 1997). Dichoptic presentation was achieved by using a 120-Hz monitor (27-inch, spatial resolution 1920 × 1080, VG278, ASUS), paired with shutter goggles (Nvidia 3D Vision 2). The experiment was performed in a darkened room. Observers’ heads were stabilized with a chinrest, 50 cm from display. The experiment spanned two sessions occurring on separate days. In the first sessions, observers were visually screened and completed all behavioral tasks. The order of the tasks was randomized. The second session constituted the MRI/DWI acquisition which was completed within three months from the date of behavioral testing.

### Stimuli and tasks

#### Binocular phase combination

**Figure 1.**
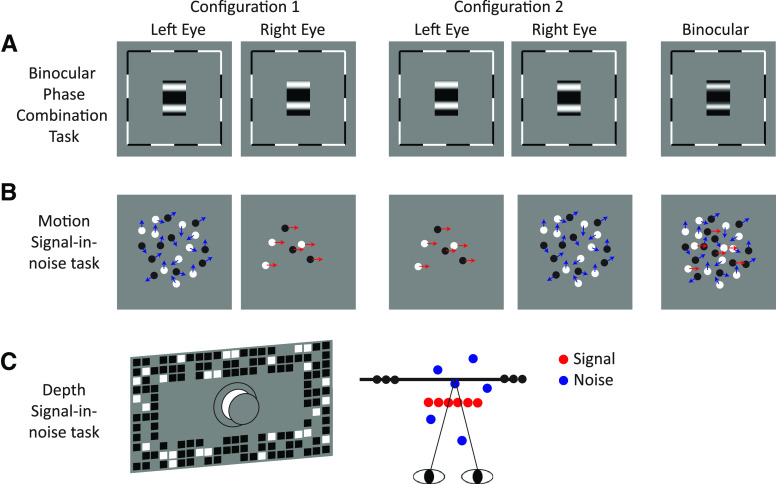
Schematics depicting (***A***) stimuli for the binocular phase combination task, (***B***) stimuli for the motion signal-in-noise task, and (***C***) stimuli for the depth signal-in-noise task.

Two horizontal sine-wave gratings (3.6° × 5.3°) with opposite vertical phase shifts (±22.5°) at 100% contrast were dichoptically presented to the two eyes (Fig. 1*A*). Spatial frequency was fixed at 0.3 cycles per degree. In a given trial, observers first viewed a fixation cross surrounded by a binocularly presented frame to aid fusion. Observers then performed calibration during which each eye was presented with a fixation cross and two diagonally positioned dots (in opposite directions for each eye). By pressing the arrow keys, observers shifted the calibration stimuli to form a single fixation cross and four symmetrically positioned dots. When satisfied with alignment, observers were then instructed to press a key to view the main stimulus. The two horizontal sine-wave gratings were dichoptically presented along with a horizontal reference line, 0.04° in thickness. Observers used up and down arrow keys to place the reference line at the perceived center of the fused cyclopean percept (perceived phase) with a fixed step size of 0.04°, corresponding to 4° phase angle of the sinewave grating. The initial position of the reference line was randomly placed within ±2° from the midline on each trial. Observers had a maximum of 120 s to adjust the reference line.

Two configurations were used to avoid positional bias. The –22.5° phase shift grating was presented randomly to the right (Fig. 1*A*, configuration 1) or left (Fig. 1*A*, configuration 2) eye on each trial. Each configuration was measured 40 times, totaling 80 trials in each block. Each observer completed one block in ∼5–6 min. The final perceived phase ( $\hat{\theta }$) is given by Equation 1. The center coordinates of the screen were defined as (0,0). Placing the reference line exactly at the center yields ${\hat{\theta }}_{C1}={\hat{\theta }}_{C2}$ = 0°, thus $\hat{\theta }$ = 0°, indicating perfectly balanced eyes. Placing the line above or below center gives negative or positive values, respectively. A negative $\hat{\theta }$ denotes right-eye-dominance. We only included responses with reaction time larger than 300 ms to filter out possible mis-clicks that prematurely ended the trial. A total of 12 trials were removed from seven observers;

(1) $$\hat{\theta }=(\frac{{\hat{\theta }}_{C1}-{\hat{\theta }}_{C2}}{2}).$$

#### Dichoptic global motion discrimination

In the two-alternative forced choice (2AFC) motion signal-in-noise (SNR) task, one eye was presented with signal dots, i.e., uniform motion direction, and the other eye was presented with noise dots, i.e., random motion direction. Signal and noise dots (0.2°, velocity 2°/s, lifetime 1 s) were composed of equal proportions of black and white dots. The stimulus was displayed on a uniform gray background with a binocularly presented frame of black and white squares (1.5°) to promote binocular fusion. Task difficulty was manipulated by changing the signal-to-noise ratio using the QUEST staircase procedure sampling thresholds at the 82%-correct level (Watson and Pelli, 1983). At 100% signal, all dots were coherently moving to the left or right. Signal dots were either presented to the right (Fig. 1*B*, configuration 1) or left eye (Fig. 1*B*, configuration 2). Each trial started with a 500-ms fixation, followed by a 500-ms presentation of the stimulus. Observers had a maximum of 1000 ms to indicate the net motion direction of the moving dots (left or right) by pressing the arrow keys. One block consisted of two randomly interleaved staircases (one per configuration), where each configuration was presented 60 times in a randomized order. Each participant completed two blocks lasting a total of ∼10 min. Motion coherence dominance ratio was given by Equation 2. We obtained the average thresholds for configurations 1 (Threshold_right_) and 2 (Threshold_left_) from both blocks. A negative motion coherence dominance ratio indicated right-eye-dominance:

(2) $$\text{Motion coherence dominance ratio}=\frac{{\text{Threshold}}_{\text{right}}-{\text{Threshold}}_{\text{left}}}{{\text{Threshold}}_{\text{left}}+{\text{Threshold}}_{\text{right}}}.$$

#### Depth signal-in-noise task

In the depth task, stimuli were random dot stereograms (RDSs) presented on a mid-gray background with a grid of black and white squares (1.5°) designed to promote stable vergence (Fig. 1*C*). The RDS consisted of equal proportions of black and white dots (0.2°, density 12 dots/deg^2^) depicting a central target (4.5° in diameter) and a surrounding annulus (9° in diameter). Observers were asked to judge whether the central target was in front (“near,” configuration 1) or behind of (“far,” configuration 2) the surround by pressing one of two arrow keys. Dots on the target plane were assigned the same disparity of ±6 arcmin and noise dots were randomly positioned within ±12 arcmin. Task difficulty was manipulated by varying the signal-to-noise ratio using the QUEST staircase procedure sampling thresholds at the 82%-correct level. At 100% signal, all dots (signal and noise) had the same disparity. On a single trial, observers viewed the 500-ms fixation cross, followed by a 500-ms presentation of the stimulus. Observers were allotted a maximum response window of 1000 ms. Each observer completed two blocks and each block consisted of two interleaved staircases (60 trials per configuration). Stereosensitivity was indexed by averaging thresholds of both configurations from the two blocks.

### MRI data acquisition and preprocessing

Neuroimaging data were acquired using a 3T Signa Premier (GE Healthcare) equipped with a 48-channel head coil at The University of Hong Kong. We collected an anatomic T1-weighted MP-RAGE image at 1-mm^3^ spatial resolution (TR = 700 ms, TE = 2.8 ms). Diffusion-weighted imaging (DWI) data were acquired using dual-spin echo planar imaging at 2-mm^3^ spatial resolution (TR = 4000 ms, TE = 73 ms). 60 20 mm-thick slices in 64 noncollinear diffusion directions (*b *=* *2000 s/mm^2^) were acquired along with two nondiffusion weighted (b = 0 s/mm^2^) volumes. The main diffusion sequence was repeated twice. We additionally obtained two reference (b = 0) images in a reversed phase-encoding direction (P-A).

Susceptibility-induced distortions were corrected using combined b0 images from A-P and P-A scans with FSL TOPUP tools (Andersson et al., 2003; Smith et al., 2004). Observers’ motion was corrected using a 14-parameter nonlinear co-registration based on an expected model of eddy-current distortions given the phase encode direction during data acquisition (Andersson and Sotiropoulos, 2016).Tools used in preprocessing are publicly available as part of MRtrix3 and FSL software packages (Smith et al., 2004; Woolrich et al., 2009; Jenkinson et al., 2012; Tournier et al., 2019).

### Fiber tractography

Using the automated segmentation procedure in Freesurfer (http://surfer.nmr.mgh.harvard.edu/; Fischl, 2012), we performed tissues segmentation on the T1 image to estimate white/gray matter boundary and locate region of interest (ROI) to serve as fiber endpoints. Specifically, we sourced the lateral geniculate nuclei (LGN; Iglesias et al., 2018), primary visual cortex (V1), fusiform, inferior temporal cortex and lateral occipital cortex using automated cortical parcellation in Freesurfer.

#### Optic radiation (OR)

OR streamlines were identified by performing probabilistic tractography between LGN and V1 based on ConTrack (Stanford University, Stanford, CA; http://github.com/vistalab/vistasoft; Sherbondy et al., 2008a). We sampled 100,000 candidate pathways connecting the LGN and V1 (step size, 1 mm) and retained the 5000 pathways with the highest likelihood based on diffusion measurements at each sampling node in the ConTrack scoring process (Sherbondy et al., 2008b). We further refined the tracts in Quench (Cassette, 2016) by removing OR tracts belonging to the bottom 2.06% for linearity, and restricting their maximum length to 115 mm to filter out feedback streamlines that resemble the template for splenium of the corpus callosum via visual inspection (Renauld et al., 2016). Detailed description of the ConTrack procedure have been described elsewhere (Sherbondy et al., 2008a, b).

#### Vertical occipital fasciculus (VOF)

We first generated the whole-brain connectome using the iFOD2 probabilistic algorithm (Tournier et al., 2010). This algorithm is reliable for delineating fibers in highly curved and crossing regions. We sampled 2 million streamlines with a maximum length of 250 mm and FOD amplitude cutoff at 0.06. Automated fiber quantification was then used to identify the VOF tracts using the whole-brain connectome and previously identified ROIs (fusiform, inferior temporal cortex, and lateral occipital cortex). Fibers that traveled at least 1.3 cm farther vertically than other directions, were located posterior to the arcuate fasciculus and had ventral endpoints near the ROIs were selected as candidate streamlines. The algorithms are publicly distributed in the VOF toolbox as part of the AFQ software package (http://github.com/yeatmanlab/AFQ; Yeatman et al., 2012). More details of VOF tractography can also be found from previous work (Yeatman et al., 2014; Duan et al., 2015).

#### Occipital corpus callosum (OCC)

OCC tracts were identified with open-source MATLAB codes from AFQ toolbox (Yeatman et al., 2012). This function tracks fibers that passed through the mid-sagittal plane of the corpus callosum and two ROIs, left and right V1 (RF Dougherty et al., 2007).

### Quantifying tissue properties

We obtained fractional anisotropy (FA) and mean diffusivity (MD) values using mrDiffusion tools in the Vistasoft package (Stanford University, Stanford, CA; http://github.com/vistalab/vistasoft). Each tract was sampled at 100 equidistant nodes with first and last 10 nodes removed to exclude voxels that are too close to the gray-white matter interface. We summarized the tract profiles using FA and MD values sampled at the central 80 nodes, averaged across two runs. We successfully obtained FA and MD values on left and right OR and OCC from all 41 observers. Because of possible motion and/or susceptibility artifacts that could not be corrected, despite successful VOF reconstruction, we obtained null FA values of left and right VOF from six and four observers, respectively. For MD, we extracted null tract left VOF means from three observers and that of right VOF from one observer. This is a common, recurring issue with the AFQ toolbox, especially when the tensor could be ill defined near cortical endpoints or in regions with artifacts.

### Statistical analysis: DWI and behavior

All statistical analyses were performed in MATLAB using functions from the Statistics and Machine Learning Toolbox. Since we were interested in whether tract properties could explain the degree but not directionality of SED, and owing to the non-normality of the data, behavioral data were first analyzed using Spearman rank correlations to investigate the possible relationships among SED metrics (absolute perceived phase and absolute motion coherence dominance ratio), and SED and stereosensitivity (% depth signal). To investigate tracts that may best predict the various behavioral SED metrics, we entered data into (Bonferroni-corrected) multiple linear regressions. Although the models are not strictly independent, we opted for full seven-way Bonferroni correction to be fully conservative. Before entering the diffusion metrics into the analyses, we derived metrics that were deemed most sensible in accordance with the tract endpoints’ known monocular or binocular status. For instance, while SED references a comparison between left and right eye inputs, it would not make a great deal of sense to compare right versus left tract tissue properties beyond V1, subsequent to known emergence of binocularity.

We derived diffusivity metrics as follows:

#### OR

Given that neurons of each of the right and left LGNs are exclusively monocular and have representations from both eyes (be it within separate layers), we elected to compute absolute weighted tract differences between left and right OR ( $\left|\frac{L-R}{L+R}\right|$) – OR_weighted_. Beyond the chiasm, white matter will not just carry information from the contralateral eye but rather have information originating from both eyes. As noted earlier, while nasal fibers from both eyes decussate, there is a significantly higher density of (i.e., 300% more) ganglion cells carrying information from the nasal retina than the temporal retina (Curcio and Allen, 1990). Beyond the chiasm then, and within each hemisphere, there is a physiological imbalance as to the white matter representations in terms of the eye of origin. For this reason, for the OR, we retained a metric that reflects L versus R asymmetry. We weighted the differences as it is well possible for individual tract/radiation differences to scale with overall tissue properties. Finally, we elected to take the “absolute” asymmetries, as the signage of the perceptual manifestation of SED is not intuitive, especially as it relates to physiological asymmetries (Dieter and Blake, 2015; Stanley et al., 2019). We arrive at a metric of the OR then, that allows us to gauge correlates of the degree of eye dominance.

#### VOF

As binocular combination occurs at V1, any difference in the weighting (or here, transmission) of the two eyes’ inputs should not be reflected in comparisons of the lateralized tracts, but rather reflected in a weakened average. Hence, VOF tract tissue properties were represented as the average of left and right VOF – VOF_average_.

#### OCC

We took the FA and MD values as they were.

To infer the stability of our estimate, we computed confidence intervals for nonintercept coefficients by drawing 1000 bootstrapped samples from residuals.

## Results

### Behavioral indices of sensory eye dominance and stereosensitivity

**Figure 2.**
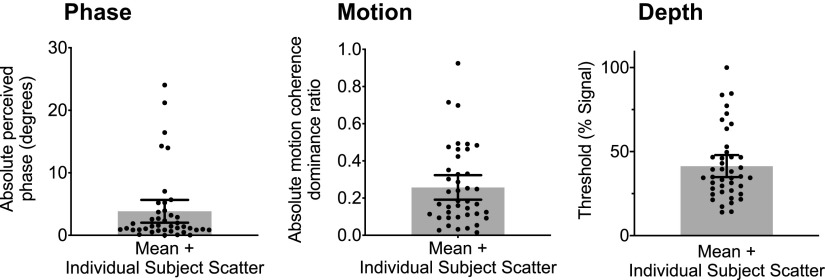
Results from the behavioral tasks. Bar+scatter plots reflecting mean absolute perceived phase and distribution (left), mean absolute motion coherence dominance ratio and distribution (middle), mean depth discrimination threshold and distribution (right). Error bars represent the 95% confidence interval.

We first examined observers’ degree of sensory eye dominance and stereosensitivity as indexed using the binocular phase combination (“phase”), dichoptic global motion discrimination (“motion”) and depth signal-in-noise tasks (“depth”; Fig. 2). Observers’ degree of sensory eye dominance was classified according to their absolute perceived phase and absolute motion coherence dominance ratio. Stereosensitivity was estimated as observers’ depth discrimination threshold (percentage of signal dots required for 82%-accurate discrimination). The lower the threshold, the greater the stereosensitivity. Overall, observers had a mean absolute perceived phase of 3.841 ± 0.898°, mean absolute motion coherence dominance ratio of 0.257 ± 0.033 and mean depth discrimination threshold of 41.349 ± 3.242 (% signal).

### Strong correlation between SED indices

**Figure 3.**
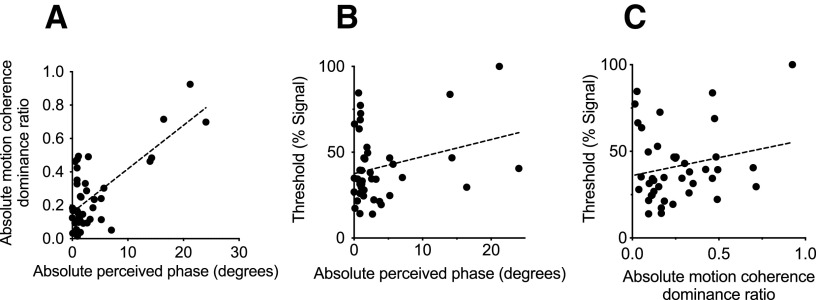
Correlations between the behavioral measures. ***A***, A positive relationship between absolute perceived phase and absolute motion coherence dominance ratio. ***B***, No relationship between absolute perceived phase and stereosensitivity nor (***C***) between motion coherence dominance ratio and stereosensitivity.

We next examined relationships among the behavioral metrices using Spearman rank correlations (corrected to hold family-wise error at 0.05). We found a significant positive relationship between the two SED (phase and motion) tasks (ρ* *=* *0.35, *p *=* *0.025; Fig. 3*A*). Neither SED indices significantly correlated with stereosensitivity [Phase: ρ* *=* *0.081, *p *=* *0.615 (Fig. 3*B*); Motion: ρ* *=* *0.071, *p *=* *0.657 (Fig. 3*C*)].

### Tract properties of ORs explain variances in SED

**Figure 4.**
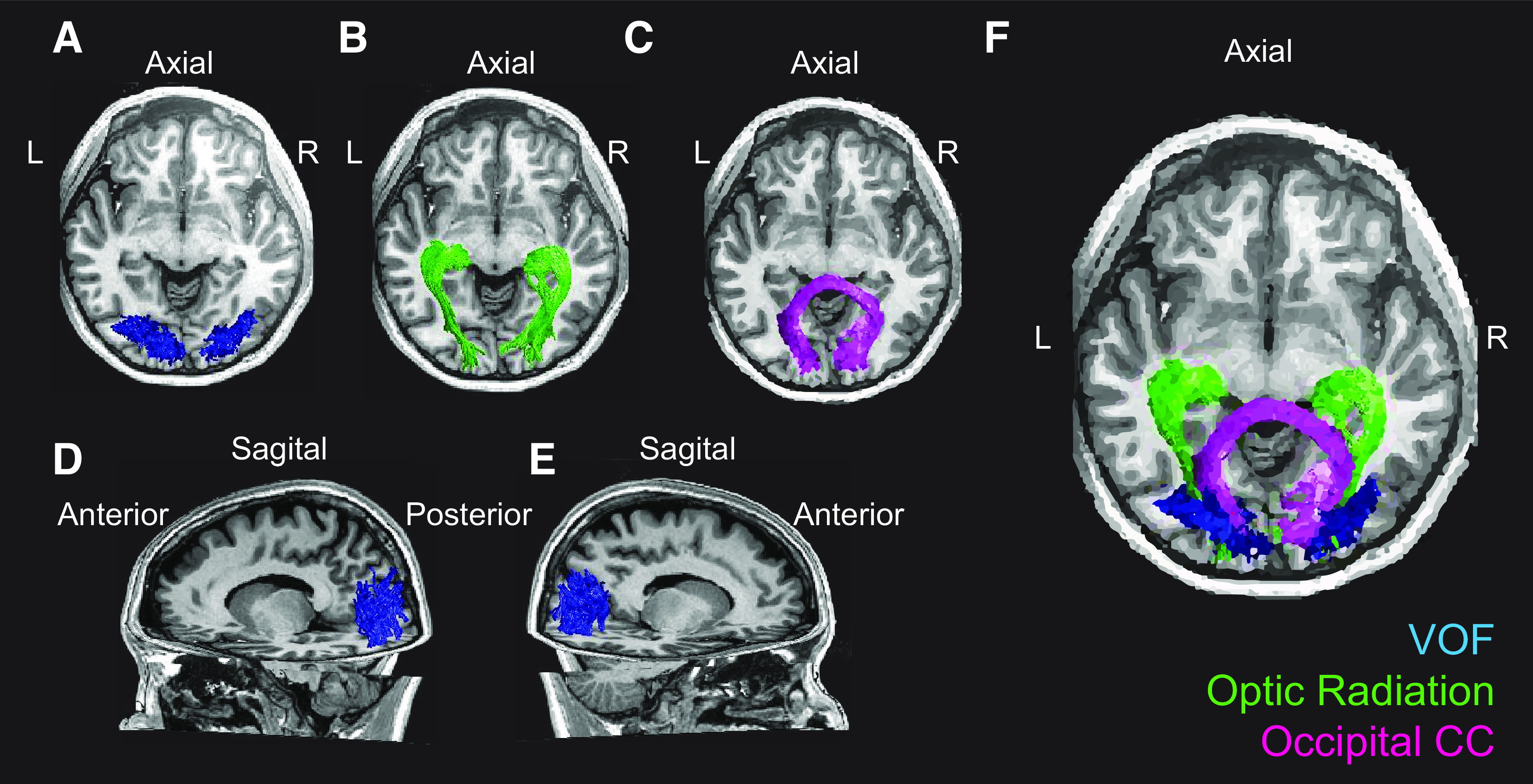
White matter tracts estimated by tractography in one representative observer. Axial views of (***A***) VOF, (***B***) OR, (***C***) OCC. Sagittal views of (***D***) left VOF, (***E***) right VOF. ***F***, Axial view of all five tracts (green, OR; magenta, OCC; blue, VOF).

To investigate how tract diffusivity measures may contribute to variations in behavioral performance, we entered FA and MD values of the tracts (Fig. 4) in multiple linear regression models to assess their predictive value to behavior.

**Figure 5.**
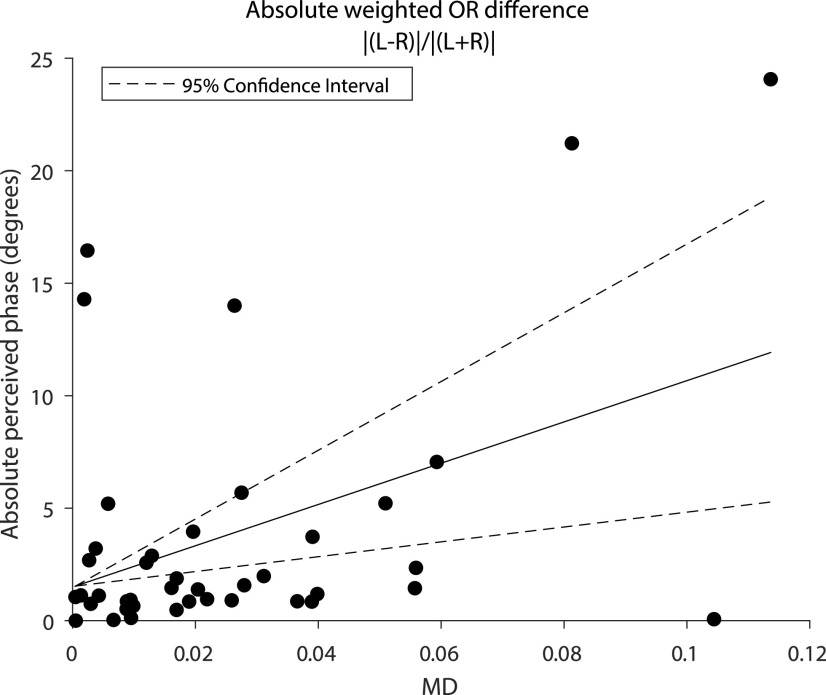
Regression fits and bootstrapped coefficient confidence interval of absolute weighted differences in OR MD values and absolute perceived phase.

For each behavioral metric, and for each of the FA and MD properties, we tested seven regression models comprising single or combinations of OR_weighted_, VOF_average_, as predictors. The multiple regressions were Bonferroni-corrected to hold family-wise error at 0.05 via a seven-way Bonferroni correction. The analyses indicated that MD values of OR_weighted_ could significantly predict perceived phase (phase; *R*^2^ = 0.185, *F*_(2,38)_ = 8.824, *p *=* *0.005; Table 1). The significance of this model is confirmed by the bootstrapped coefficient confidence interval as it does not include 0 (Fig. 5). MD values of OR_weighted_ + OCC also predicted, to a weaker extent, perceived phase although these models did not survive the conservative seven-way statistical correction (*R*^2^ = 0.212, *F*_(3,37)_ = 5.117, *p *=* *0.01; Table 1). Neither FA nor MD could predict SED as indexed by the motion coherence dominance ratio (SED motion) nor stereosensitivity (depth).

**Table 1.** Statistical evaluation of multiple linear regression models

Task	Variables in the model	Tract property	*R* ^2^	*p*
SED phase	OR_weighted_	FA	0.034	0.251
		MD	0.185	0.005*
	VOF_average_	FA	0.004	0.722
		MD	0.003	0.735
	OCC	FA	0.051	0.158
		MD	0.010	0.533
	OR_weighted_, VOF_average_	FA	0.031	0.642
		MD	0.196	0.025
	OR_weighted_, OCC	FA	0.068	0.265
		MD	0.212	0.011
	VOF_average_, OCC	FA	0.050	0.490
		MD	0.020	0.711
	All tracts	FA	0.063	0.615
		MD	0.225	0.036
SED motion	OR_weighted_	FA	0.000	0.955
		MD	0.067	0.102
	VOF_average_	FA	0.008	0.632
		MD	0.013	0.504
	OCC	FA	0.010	0.530
		MD	0.002	0.803
	OR_weighted_, VOF_average_	FA	0.009	0.879
		MD	0.075	0.266
	OR_weighted_, OCC	FA	0.010	0.818
		MD	0.067	0.267
	VOF_average_, OCC	FA	0.03	0.655
		MD	0.013	0.797
	All tracts	FA	0.030	0.842
		MD	0.075	0.456
Depth	OR_weighted_	FA	0.001	0.870
		MD	0.066	0.106
	VOF_average_	FA	0.086	0.110
		MD	0.026	0.340
	OCC	FA	0.006	0.643
		MD	0.023	0.347
	OR_weighted_, VOF_average_	FA	0.093	0.255
		MD	0.070	0.289
	OR_weighted_, OCC	FA	0.008	0.863
		MD	0.102	0.129
	VOF_average_, OCC	FA	0.111	0.194
		MD	0.074	0.272
	All tracts	FA	0.127	0.293
		MD	0.128	0.205

*p ≤* *0.007, **p ≤* *0.001, ***p ≤* *0.00001.

## Discussion

Exploiting variations in sensory eye dominance in the neurotypical population, we examined whether major visual white matter tract properties could explain individual variabilities in SED. We found that indices from the two SED tasks (binocular phase combination and dichoptic global motion discrimination tasks) strongly correlated with each other. However, neither SED index correlated with stereosensitivity as measured by the depth signal-in-noise task. Examining the microstructural properties of major visual white matter tracts, left and right OR, left and right VOF, and OCC, we found that only regression models involving MD values of the left and right ORs (and to a weaker extent, OCC) could predict SED as indexed by perceived phase. Neither MD nor FA could predict performance on the dichoptic motion coherence task, nor on the stereo-depth task. These findings suggest that SED is reflected, at least in part, by distributed tract properties along early (thalamocortical) and late cortical visual pathways. The lack of association between each of the motion coherence dominance, and stereosensitivity metric with tract properties suggest a possible neural dissociation between the phase and motion tasks – such that while they may both be effective metrics of SED, the latter may be governed by factors in addition to/other than microstructural properties of visual white matter tracts.

### SED tasks are behaviorally correlated

Our psychophysical data are in good agreement with previous studies showing that SED measured with phase and motion stimuli are largely consistent in terms of magnitude (Han et al., 2018; Ooi and He, 2020; Fig. 3*A*). Notably, the two SED tasks involve the presentation of distinct features: the phase task presents a pair of static, fusible horizontal gratings and the motion task presents dichoptically, moving signal and noise dots. The binocular phase combination task then, is likely suited to orientation selective channels in area V1 (Huang et al., 2010; Zhou et al., 2013) while the dichoptic motion task likely suits motion-sensitive mechanisms in the human middle temporal complex (hMT^+^; Beckers and Hömberg, 1992). Beyond the featural differences, the strong correlation between the SED tasks indicates that the two processes engage in common circuitry at some stage. One possible location of interaction is the LGN, which receives cortical projections from V1, where V2, V4 and MT provide strong feedback to V1 (Fitzpatrick et al., 1994; C Wang et al., 2007; Briggs et al., 2016). Cortical feedback may modulate gain-control signals and the strength of the inhibitory network at the LGN.

### No relationship between SED and stereosensitivity

We did not find correlations between SED and stereosensitivity (Fig. 3*B*,*C*). This is somewhat counter to reports from several earlier studies (Dieter et al., 2017; Han et al., 2018), but are in agreement with Y Wang et al. (2018), who found no correlation between SED as measured by the binocular phase combination task and stereopsis, as well as Wu et al. (2018), who indexed SED by means of the continuous flashing technique, and similarly did not find any relationship of SED with stereosensitivity. The lack of correlation observed is perhaps not surprising, as we indexed SED and depth sensitivity from the neurotypical population, whose within-subject variability is relatively minimal compared with the clinical population, e.g., amblyopia or other age groups, e.g., elderly. Moreover, certainly, stereopsis entails the participation of binocular neurons outside of V1, but also along key disparity-sensitive nodes such as V3 and V7 (Tsao et al., 2003; Brouwer et al., 2005). Indeed, Wu et al. (2018) suggested that SED affects the rate of signal integration, while stereoacuity depends on the efficiency of binocular neurons on extracting disparity information from the integrated signals, which should be independent from SED. This would also be consistent with findings of Feng et al. (2017), who found that treated anisometropes still exhibited strong SED as indexed by the binocular phase combination task, but had near to neurotypical stereoacuity indexed by clinical tests. Thus, it seems that SED alone is insufficient to explain stereoscopic outcomes.

### The neural correlates of SED

We found that absolute weighted differences of MD values of the optic radiations are well associated with the magnitude of SED indexed by the phase task (Table 1; Fig. 5). These findings suggest that SED is at least in part explained by white matter microstructural properties early in the visual cascade and well fit the gain-control theory (Ding and Sperling, 2006) and particularly, the speculative interocular contrast gain control mechanisms placed before binocular summation. The variability in perceived phase may be associated with different levels of myelination in the left and right ORs that are then reflected by MD. However, prominent to Figure 3*A* is the presence of several cases of strong eye dominance (paired with larger phase indices). If these individuals are removed, the correlation (*p* = 0.005) no longer survives a strict seven-way statistical correction. For this reason, while exciting, we caution that the relationship between optic radiation tissue properties and SED will require future empirical verification.

We note here that white matter microstructural properties of the ORs have been previously shown to relate to gray-matter visual function. Specifically, Toosy et al. (2004) reported a positive relationship between white matter microstructural properties (in terms of FA values) of ORs and BOLD activity in the visual cortex. More relevant to the present work, underdevelopment of the ORs has been observed in amblyopes (Xie et al., 2007; Qi et al., 2016) who display extreme forms of eye imbalance. Since ORs carry information bidirectionally, variability in MD values may lead to changes in both the LGN and V1. Indeed, Qi et al. (2016) reported a positive relationship between OR tissue properties and cortical thickness of V1, implying that the impairment of ORs may be associated with structural deficits in the visual cortex. It follows that any structural gray matter deficits in V1, or potentially even earlier in the LGN, may reduce the efficiency in the processing of visual information, with efficiency here loosely encapsulating both readouts and binocular interactions, eventually manifesting in binocular imbalance.

It is worth noting that our data do not preclude the possibility that there are roles for further tracts that occur “postsummation” (i.e., post-V1; Baker and Meese, 2007; Ding et al., 2013; Ding and Levi, 2017). We note the potential involvement in our data of the OCC as it relates to SED, although the model did not survive the full statistical corrections (Table 1). While the exact relationship of the OCC to SED needs to be further validated, it is interesting to speculate on its role given its anatomic placement of connecting the visual cortex in left and right hemispheres. Interhemispheric processing has been studied in the rat visual cortex where the rodent is monocularly deprived during the critical period of visual development (Pietrasanta et al., 2012). By silencing the callosal pathway, Pietrasanta et al. (2012) managed to reduce the deprivation-induced shift in ocular dominance and rats were able to regain binocularity once the deprived eye started receiving visual input. It appears that OCC is key to developmental maturation of the two hemispheres and neurotypical binocularity. Perhaps more efficient neuronal transmission between the two hemispheres may facilitate binocular integration.

### Microstructural properties of visual white matter tracts could not predict performance in the dichoptic motion task

While tract properties seem to well-predict SED as indexed by the phase task, the same was not true of the dichoptic motion task SED metric. While at first glance this might be surprising, our motion task, unlike the V1-oriented phase-combination-task, taps into features known to be extrastriate-reliant, e.g., human middle temporal complex hMT^+^ (Beckers and Hömberg, 1992) and posterior parietal cortex (Patten and Welchman, 2015). The optic radiations connect LGN to V1 and VOFs have their endpoints in fusiform, inferior temporal cortex and lateral occipital cortex. Motion signal extraction, as required by the dichoptic motion task, seems to be largely determined by hMT^+^ neurons’ ocular preference. hMT^+^ neurons are mostly (45–60%) binocularly unbiased or balanced, with the rest showing clear ocular dominance (Maunsell and Van Essen, 1983; Kiorpes et al., 1996; El-Shamayleh et al., 2010). The ocular-specific responses motion neurons render a motion-oriented task quite suitable for picking up eye imbalances, but these imbalances should be reflected in terms of hMT+ cortical responses. That is, SED imbalances as reflected by the dichoptic motion task may be better captured by high-resolution functional gray matter responses, rather than by solely microstructural properties of white matter.

### Outstanding issues

We close with two outstanding issues that may be worth empirical attention moving forward. The first relates to the fact that many aspects of our data are null (e.g., lack of correlation between SED and certain visual white matter tract properties, lack of correlations between SED and stereosensitivity). Still, we believe that there is still value in reporting this information to the community to help weigh in on prevailing theories on the neural basis of SED. While it is intriguing that the microstructural properties of the optic radiations are a strong predictor of behavioral performance in the phase task, these positive results should be reported with caveats, the possibility that certain correlations could not be detected because of the sensitivity limitations of the techniques, i.e., imaging resolution, reliability of SED tasks.

Moreover, in our pool of neurotypical participants, there is a lack of consistency in eye dominance classifications. In only a little over half of our participants (53.7%) was there eye dominance agreement across both SED indexing tasks. This observation, however, is not surprising in light of previous work demonstrating comparably weak consistency across standard SED tasks when indexing eye dominance in the neurotypical population (García-Pérez and Peli, 2019; Kam and Chang, 2021). If we look only at the subset of our participants with strong eye dominance (2 SD beyond mean), we find that they show high consistency across both SED tasks. We speculate that the inconsistency among eye dominance classifications may be because of the relatively balanced eyes in neurotypical individuals, thus classifications for weakly dominant eyes come down to variance. Yet, it remains an open question as to whether alternate SED indices (i.e., binocular rivalry) may be more sensitive to revealing functional relationships with white matter, as well as with broader binocular function (i.e., stereosensitivity).

In conclusion, the present data suggest that SED is governed at least in part by properties of the optic radiations. Although both dichoptic phase and motion tasks are well-established behavioral metrics of SED, the two tasks may yet capture distinct neural mechanisms, with the latter better characterized by gray matter function. Our data further assert that SED alone is insufficient to predict stereoscopic function.

## Synthesis

Reviewing Editor: Anne Keitel, University of Dundee Decisions are customarily a result of the Reviewing Editor and the peer reviewers coming together and discussing their recommendations until a consensus is reached. When revisions are invited, a fact-based synthesis statement explaining their decision and outlining what is needed to prepare a revision will be listed below. The following reviewer(s) agreed to reveal their identity: Hiromasa Takemura.

Please see below for Reviewer #2s comments on the revised version of your manuscript. Unfortunately, Reviewer #1 was not available to assess the revised manuscript. However, the available reviewer and I both thought the revisions were well done and caveats/open questions were highlighted sufficiently.

Reviewer #2 - Advances the Field (Required)

This manuscript reports a novel examination on correlation between sensory eye dominance and tissue properties of the optic radiation. I think that this is a new research question which will motivate future studies. Only issue is that the effect is not large. However, considering that this is a journal which publishes carefully conducted negative results, I think that it is fine to publish this manuscript since authors acknowledge that effect size is limited.

Reviewer #2 - Statistics

Authors made a response to regression models and it seems OK to me, but I am not really confident on this point.

Reviewer #2 - Comments to the Authors (Required)

I thank authors to carefully address all my comments. I have no additional comments.
